# Wiedemann-Steiner syndrome: description of genetic profiles and clinical phenotypes of 10 Korean pediatric patients

**DOI:** 10.1186/s12920-025-02226-0

**Published:** 2025-10-06

**Authors:** Jeesun Yoo, A Young Park, Jung Min Ko

**Affiliations:** 1 Department of Pediatrics, Seoul National University College of Medicine, 101, Daehak-ro, Jongno-gu, Seoul, Korea; 2 Department of Pediatrics, Hallym University Sacred Heart Hospital, Anyang-si, Gyeonggi-do Korea; 3 Rare Disease Center, Seoul National University Hospital, Seoul, Korea

**Keywords:** Wiedemann-Steiner syndrome, *KMT2A*, Developmental delay, Hypertrichosis, Growth retardation

## Abstract

**Background:**

Wiedemann-Steiner syndrome (WSS) is a genetic malformation syndrome caused by abnormalities in *KMT2A*. It is characterized by developmental delays, facial dysmorphism, hypertrichosis, failure to thrive, and musculoskeletal anomalies. Expanded applications of exome sequencing have increased the number of confirmed cases, broadening our understanding of the WSS spectrum.

**Methods:**

We collected and organized the clinical and molecular features of 10 unrelated Korean patients diagnosed with WSS using molecular analysis. Clinical characteristics were presented based on the electronic medical records of the patients’ regular visits.

**Results:**

Subject patients consisted of four male and six female patients. The median patient age at diagnosis was 6.76 years. In most cases, the chief complaint upon visiting a clinician was developmental delay (8/10). The most frequently observed phenotypes included failure to thrive (9/10), short stature (7/10), developmental delay (10/10), and hypertrichosis (10/10). The degree of developmental delay varied among the patients. The majority (9/10) were diagnosed by exome sequencing, with the exception of one patient (1/10) who had a microdeletion at 11q23.3, encompassing partial *KMT2A*, as diagnosed by chromosomal microarray. All patients had private pathogenic or Likely pathogenic variants without any recurrent variants, and nine of the 10 variants were novel.

**Conclusions:**

Most Korean patients with WSS exhibited suggestive features, but most were not pathognomonic of WSS; thus, many patients may only be identifiable by molecular analyses. Phenotypes frequently overlap with other chromatinopathy syndromes. Future studies are needed to determine the genetic background of patients with molecularly unresolved WSS and to further delineate WSS.

## Introduction

Wiedemann-Steiner syndrome (WSS), first described in 1989 by Wiedemann et al., is a genetic disorder characterized by developmental delay accompanied by distinctive facial dysmorphisms. WSS is caused by monoallelic pathogenic variants, and the causative gene was identified as *KMT2A* in 2012 [1]. *KMT2A* encodes histone methyltransferases, a DNA-binding protein, and KMT2A methylates the lysine 4 on histone 3 (H3K4) [2, 3]. This protein is involved in the regulation of the chromatin-mediated transcription of multiple genes, including the expression of *Hox* and *Wnt* family factors. Histone modifications, such as methylation, have been suggested to play an important role in neurogenesis, and abnormal histone lysine methylation can lead to impaired human intellectual development [4, 5].

In addition to cognitive development, WSS is characterized by distinctive phenotypic features, such as growth retardation, facial features, hypertrichosis cubiti, and skeletal deformities, such as clinodactyly or craniovertebral junction anomalies. Some patients present with cardiac anomalies, ophthalmological anomalies, urogenital anomalies, and psychiatric disorders. In addition, rare cases accompanied by immunodeficiency have been reported [6, 7]. The long-term clinical course has not been elucidated in detail since almost 250 cases have been reported in the literature [1], and only a few adolescent and adult patients have been included [8, 9]. Accordingly, there are limited data on the overall prognosis of various phenotypes involving each organ system or the life expectancy of patients.

Few studies have been conducted on patients in East Asia. The largest cohort study of 104 participants included only six Asian patients, and there have been only two case reports, including three patients in Korea [1, 10, 11]. Here, we investigated the clinical and genetic characteristics of 10 Korean patients with WSS, making it the largest Korean WSS cohort to date.

## Materials and methods

### Subjects

A total of 10 patients (four males and six females) from 10 unrelated families were diagnosed with WSS by molecular genetic analyses between 2015 and 2020 and were enrolled in this study. Data on clinical phenotypes were collected through a retrospective review of the electronic medical records at Seoul National University Children’s Hospital.

### Clinical information

World Health Organization (WHO) growth curves were used for growth parameter analysis such as height, weight, and head circumference. It was defined as failure to thrive when the weight was less than the 5th percentile of two or more anthropometric measurements. Short stature was defined as a height less than the 3rd percentile, and microcephaly was defined as a head circumference less than the 3rd percentile in at least one anthropometric measurement.

The presence of developmental delay and/or muscle hypotonia was judged by a pediatric neurologist and a clinical geneticist after careful history taking and examination. Some patients underwent brain magnetic resonance imaging (MRI).

### Genetic analysis

Conventional karyotype analysis using peripheral leukocytes was previously performed in four of 10 patients, but all four patients showed a normal karyotype. Genomic DNA was extracted from nucleated peripheral blood cells collected from all 10 patients using a DNA isolation kit (Qiagen, Hilden, Germany). In eight patients, including four patients who previously showed a normal karyotype by conventional karyotyping, chromosomal microarray analysis (CMA) was performed as the first step for diagnosis. For nine patients, excluding one diagnosed with WSS by CMA, exome sequencing (ES) was additionally performed as the second step. The experimental processes were performed as described previously, with minor modifications [12]. All variants were filtered according to their predicted effects on the protein and population frequencies. Finally, we assessed the identified variants as pathogenic or likely pathogenic based on the American College of Medical Genetics and Genomics (ACMG) and Association for Molecular Pathology guidelines [13]. After the causative variant was identified from ES, it was validated by Sanger sequencing in all patients and their parents who allowed.

## Results

### Clinical characteristics

The study participants comprised four males and six females. The median age at diagnosis of the patients was 6.76 years [range 1.58–11.51], and the median follow-up period was 4.99 years [range 4.55–9.74]. The patients’ clinical characteristics are summarized and organized in Table 1.

**Table 1:** Details of clinical characteristics of 10 Korean patients with WSS

Patient	C-01	C-02	C-03	C-04	C-05	C-06	C-07	C-08	C-09	C-10	SNUCH (*n* = 10)	Cohort of 104 individuals - Sheppard et al. (*n* = 104)
Chief complaint	microcephaly	DDH, facial dysmorphism	DD	DD, facial dysmorphism, short stature	DD	DD, short stature	DD	Facial dysmorphism, DD	DD	DD		
Sex (M: F)	F	F	M	F	F	F	M	F	M	M	4:6	52:52
Age at presentation (years)	0.7	0.5	8.2	11.5	1.0	8.6	0.7	1.0	3.1	6.1		
Age at initial anthropometric data (years)	0.7	2.1	8.2	11.5	4.3	8.6	0.7	1.0	3.3	6.1		
Height at initial anthropometric data (cm, SDS)	62 (−2.86)	80.6 (−1.83)	115.9 (−2.57)	132.7 (−2.47)	87 (−4.24)	122.9 (−1.41)	69 (−0.72)	73.6 (−0.15)	85 (−3.93)	121 (0.96)		
Weight at initial anthropometric data (kg, SDS)	5.9 (−2.31)	9.9 (−1.37)	21.1 (−1.94)	32 (−1.4)	12 (−3.47)	27.45 (−0.25)	7 (−1.89)	7.5 (−1.46)	9 (−5.59)	21 (−0.2)		
Head circumference at initial anthropometric data (cm)	41.4	44.5	49.5	49.8	46				46	49.8		
Gestational age at birth	37 + 2	40 + 3	38 + 2	40	38 + 2	37 + 3	34 + 0	39	38	37	Preterm birth 10% (1/10)	
Birthweight (kg)	2.8	2.7	2.76	3.35	2.6	2.384	2.7	3.005	2.4	2		
Cesarean section, Reason	+	-	+	-	+, fetal deceleration	+, PROM	-	-	-	+, previous C/S		
NICU care, Reason	-	+, inflammatory marker elevation	-	-	+, perinatal distress, MAS	-	-	+, MAS	-	+, LBWI, neonatal jaundice	40% (4/10)	
Growth profile												
FTT	+	+	+	+	+	+	+	+	+	-	90% (9/10)	67.7%(67/99)
Feeding difficulty (Tube feeding)	+ (-)	+ (-)	- (-)	- (-)	+ (+)	- (-)	+ (-)	- (-)	- (-)	- (-)	40% (4/10)	66.3% (67/101)
Short stature	+	+	+	+	+	-	+	-	+	-	< 3p, 70%(7/10)	< 5p, 57.8%
GH deficiency		-	-		-						0%(0/3)	18.8% (6/32)
Facial dysmorphism												
Ptosis	-	+	-	-	+	+	+	-	-	-	40%(4/10)	43%(40/93)
Low set ears	-	+	-	-	+	-	-	+	-	-	30%(3/10)	
Low hairline	+	-	+	-	-	-	-	-	-	-	20%(2/10)	
Thick/long eyebrow	-	-	-	+	-	-	-	+	-	-	20%(2/10)	75.5%(77/102)
Flat nasal bridge	-	+	-	-	-	-	-	-	-	-	10%(1/10)	Wide nasal bridge 63.4% (64/101)
Others	+, small mouth	+, short palpebral fissures, short neck, high arched palate		+, dental malocclusion, narrow forehead				+, down-slanted palpebral fissures	+, coarse face, wide spaced teeth			
Neurodevelopmental & psychiatric											DD/ID 100% (10/10)	DD/ID 97% (96/99)
Congnitive ability test	NA	FSIQ 50-70 class 3 ID	NA	FSIQ 50–70 class 3 ID	FSIQ 45 class 2 ID MBI 15	FSIQ 50-70 class 3 ID	NA	NA	NA	NA		
Microcephaly	+	+	-	-	+	-	-	-	+	-	< 3p, 40%(4/10)	< 5p, 34.6%
Seizure with AED medication	+	-	-	-	-	-	-	-	+	-	20%(2/10)	20%(15/75)
Psychologic & behavioral problem	-	-	+, ADHD	-	+, insomnia, aggressive behavior	-	-	-	-	-		Sleep problem, 46.2%(42/91) Aggressive behavior, 33%(31/94) Autism, 21.3%(20/94)
Age at walking	-	15 months	16 months	12 months	35 months	24 months	32 months	18 months	30 months	14 months	Median, 18mo [12-32mo]	Median, 20mo [11-60mo]
Lastest description of developmental stage	steel creep at 5 years old, recognizes parents and calling name, but generally poor receptive languages	ordinary class of elementary school with peers of same age, difficulties in certain subjects such as math	special class of ordinary elementary school, on language rehabilitation	special class of ordinary elementary school	special education school, everyday life requires full assistance of others	special class of ordinary elementary school	1 year delay of entrance to school, special class of ordinary elementary school	attending alternative school, on language rehabilitation	no meaningful word expression at 4 years old, severe developmental retardation & arrest	expression of 20 words but no sentence, and untrained for toilet defecation at 6 years old		
Brain MRI finding	R/O microcephaly with simplified gyral pattern	normal	normal, MRI sella		Severe thinning of corpus callosum, midbody and isthmus	normal, MRI sella	normal		Mild hypoplasia of genu and splenium of corpus callosum	r/o delayed myelination of both extreme capsule and temporal lobes	Brain MRI abnormality, 50%(4/8)	Brain MRI abnormality, 57.5%(30/52)
Cardiac												
Congenital heart defect, type	+, PDA	+, PDA	-	-	+, PDA, mild AS, dysplastic aortic valve	-	-	-	-	-	3/10 (30%)	28.4%(23/81)
Ophthalmic												
Strabismus, treatment	-	+, Occlusion therapy	-	-	+, BLR recession operation	-	+, BLR recession operation	-	-	-	30%(3/10)	37.5%(36/96)
ENT & pulmonary												
Tonsil & adenoid hypertrophy	-	-	-	-	-	-	-	+	-	-	10%(1/10)	17.3%(14/81)
Bifid uvula	-	-	-	-	-	-	-	+	-	-	10%(1/10)	7.9%(7/89)
Gastrointestinal												
Constipation	-	-	+	-	-	-	+	-	-	-	20%(2/10)	63.8%(60/94)
GER	+	-	-	-	+	-	-	-	-	-	20%(2/10)	
Musculoskeletal												
Finger anomaly, type	+, 5th finger clinodactyly	+, 5th finger clinodactyly and middle phalanx shortening	-	+, 5th finger clinodactyly	+, 5th finger mid phalanx cubbing, brachydactyly	-	+, partial 2-3th syndactyly	-	+, 5th finger clinodactyly	-	60%(6/10)	2nd −3rd toe syndactyly, 7.4%(7/94)
Sacral dimple	-	+	+	-	-	-	-	-	+	+	50%(5/10)	50.5%(46/91)
Hip dysplasia	-	+, Pavlik harness	-	-	+, Pavlik harness	-	-	-	-	-	20%(2/10)	4.8%(5/104)
Others		+, torticolis, plagiocephaly	+, cubitus valgus		+, habitual dislocation of patella & radial head							
Skin												
Hypertrichosis	+	+	+	+	+	+	+	+	+	+	100%(10/10)	Cubiti, 57.3% (55/96) Back, 67% (65/97) Lower Limbs, 46.2% (43/93)

“+” indicates the presence of a phenotype; “−” indicates its absence; blank cells represent missing or unavailable data

*AED* Anti-epileptic drug; *AS* Aortic stenosis; *BLR* Bilateral lateral rectus; *C/S* Cesarean section; *DD* Developmental delay; *DDH* Developmental dysplasia of the hip; *ENT* Ear nose throat; *FSIQ* Full-Scale Intelligence Quotient; *FTT* Failure to thrive; *GER* Gastroesophageal reflux; *GH* Growth hormone; *ID* Intellectual disability; *LBWI* Low birth weight infant; *MAS* Meconium aspiration syndrome; *MBI* Modified Barthel Index; *MRI* Magnetic resonance imaging; *NA* Not assessed; *PDA* Patent ductus arteriosus; *PROM* Premature rupture of membrane; *SDS* Standard deviation score; *SNUCH* Seoul National University Children’s Hospital

The majority of patients (9/10) were born at term of gestational age (≥ 37 + 0 weeks), and one patient was born at 34 weeks of gestational age due to premature rupture of the amniotic membrane. Half of the patients (5/10) were born via cesarean section. The documented reasons for cesarean section were fetal deceleration, premature rupture of the membrane, and history of cesarean section. Four patients required immediate admission to the Neonatal Intensive Care Unit (NICU) after birth. The reasons for NICU admission were meconium aspiration syndrome in two patients, perinatal distress in one, and low birth weight and neonatal jaundice in the other.

Most patients show one or more facial dysmorphisms suggestive of genetic syndromes. The most frequent morphological characteristic was ptosis, which occurred in four patients. The second most frequent feature was a low-set ear (3/10). Two patients had low hairlines, and two patients had thick or long eyebrows. Other characteristic features include a generally coarse face, short palpebral fissures, downslanted palpebral fissures, flat nasal bridge, narrow forehead, small mouth, short neck, high-arched palate, widely spaced teeth, and severe dental malocclusion.

**Fig. 1:**
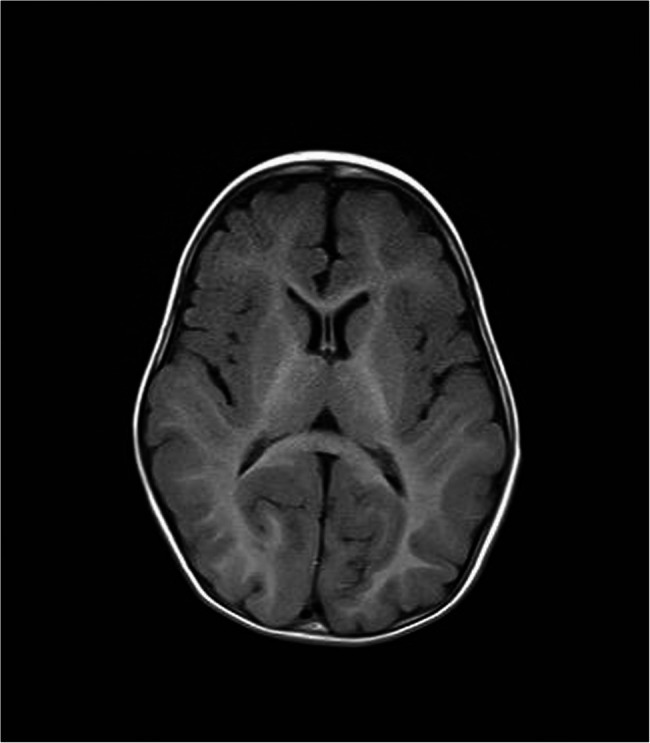
Axial view of brain MRI from patient C-01

All 10 patients presented developmental delays or intellectual disabilities (DD/ID). A history of infantile hypotonia was documented in two patients, and unprovoked seizures were observed in the other two patients. The age of walking alone varied from 12 to 35 months. Microcephaly was detected in four patients. Eight of the ten patients underwent brain MRI, and four of them did not show any specific abnormalities. In two patients who started walking alone late (each at 30 and 35 months of age), thinning of the corpus callosum was noted. One patient who could not walk alone at 5 years of age showed a simplified gyral pattern on MRI (Fig. 1). In five patients with intellectual ability tests, three of them were classified as having class 3 ID (Full-Scale Intelligence Quotient (FSIQ) 50–70), one as class 2 ID (FSIQ 35–50), and the other showed marginal intelligence. No patient met diagnostic criteria for autism spectrum disorder. Psychological and behavioral problems were observed in two patients: one was diagnosed with attention deficit hyperactivity disorder, and the other had been medically treated for insomnia and difficulty in anger control.

In terms of congenital heart defects, patent ductus arteriosus (PDA) was identified in three patients, and spontaneous closure of PDA was observed in two of them. One patient with PDA underwent device closure intervention at approximately 18 months of age. Aortic stenosis due to a dysplastic aortic valve with thick and redundant leaflets was observed in one patient.

Ophthalmic abnormalities were found in four patients. Ptosis was observed in four patients, of whom three also presented with concomitant strabismus. Two of the three patients with strabismus underwent surgical correction. The other patient was treated with glasses for hyperopia.

Musculoskeletal anomalies were frequently observed in the patients. The most common feature was finger anomaly, which was noted in six patients. Fifth finger clinodactyly was most common (four patients). Brachydactyly, partial 2nd −3rd finger syndactyly, and 5th finger middle phalanx shortening were also observed. Five patients had sacral dimples requiring spinal sonography; however, all had simple dimples without nervous system abnormalities. Two patients had developmental dysplasia of the hip; both were treated with Pavlik harness application. One patient had muscular torticollis, and surgical correction was performed. Cubitus valgus deformity and habitual dislocation or subluxation of the right elbow and knee joints were observed in one patient each.

All 10 patients exhibited hypertrichosis, which is typical of WSS. However, the area of hypertrichosis varied among patients. Hypertrichosis is not limited to the elbows but tends to be generalized, including more than two body parts. Two patients also had small hypoplastic fingers or toenails.

Some anomalies were observed in the otorhinolaryngological and pulmonary systems. One patient was incidentally found to have a tracheal bronchus deformity on chest computed tomography while she was admitted to the pediatric intensive care unit for 1 month due to viral bronchiolitis. Another patient had bifid uvula and velopharyngeal insufficiency and underwent surgical correction. Regarding the gastrointestinal system, two patients had a history of constipation, and another two patients had a history of feeding difficulty due to gastroesophageal reflux.

Failure to thrive was observed in nine of 10 patients. The parents of four patients complained of poor feeding. Seven of 10 patients also had short stature, and a growth hormone stimulation test was conducted on three of them, revealing that none of them had growth hormone deficiency, although these three patients received growth hormone therapy to achieve an increase in adult height.

### Molecular profiles

**Fig. 2:**
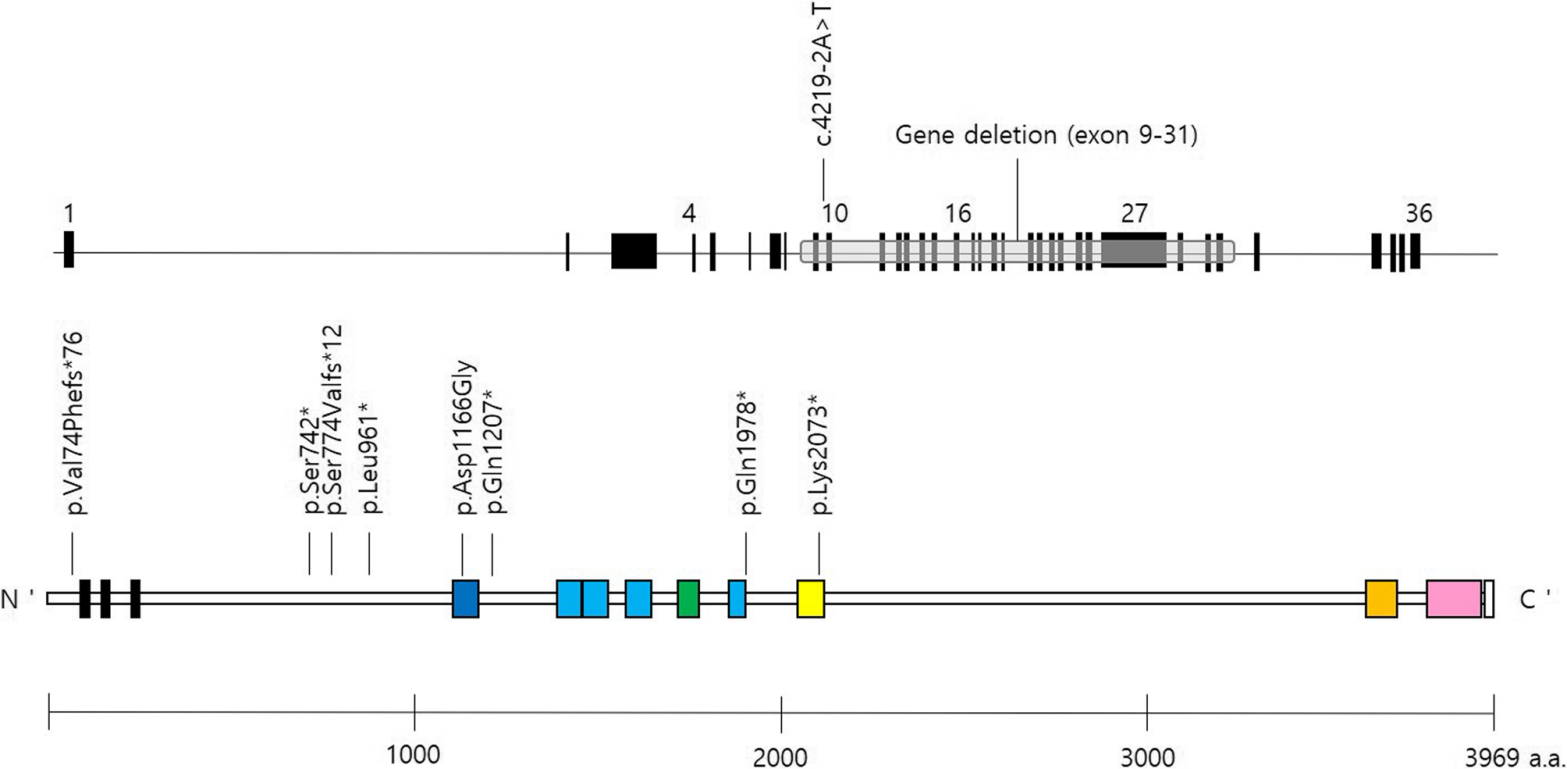
Exon structure of *KMT2A* gene [above], protein structure [below], and location of *KMT2A* mutations. Written panel shows the mutations identified in this study. Colored boxes show specific domains: black, AT hook domain; dark blue, CXXC-type zinc-finger domain; light blue, PHD-type zinc-finger domain; green, BROMO domain; yellow, FYR N-terminal domain; orange, FYR-C terminal domain; pink, SET domain; white, post-SET domain. The base positions correspond to NM_001197104.2. The domains were annotated according to the UniProt database (https://www.uniprot.org/uniprotkb/Q03164/entry#family_and_domains)

All patients were found to have pathogenic or likely pathogenic variants or a partial gene deletion in *KMT2A* (Table 2). Nine of the 10 patients were diagnosed using ES, and the remaining patient was diagnosed using CMA. Five nonsense variants: p.Ser742*, p.Gln1978*, p.Lys2073*, p.Leu961*, and p.Gln1207*. Two were frameshift variants: p.Val74Phefs*76 and p.Ser774Valfs*12. p.Ser774Valfs*12 was previously reported in 2018 [2]. Additionally, one mis-splicing variant (c.4219–2 A > T) and one missense variant (p.Asp1166Gly) were identified. One large partial gene deletion (10%) encompassing exons 9–31 of the 36 exons in *KMT2A* was detected (grch37:11:118354408–118385822). The structures of the genes and proteins, with annotations of the identified mutations, are shown in Fig. 2.

**Table 2:** Molecular profiles of 10 Korean patients with WSS

Case No	Sex	Gene	cDNA change*	Amino acid change		Type	Location		Inheritance pattern	Zygosity	Novelty	ACMG classification	ACMG classification evidence
1	F	KMT2A	c.2223dup		p.Ser742*	nonsense			*de novo*	hetero	novel	P	PVS1 + PM2 + PM6 + PP3 + PP4
2	F	KMT2A	c.5932 C > T		p.Gln1978*	nonsense			*de novo*	hetero	novel	P	PVS1 + PM1 + PM2 + PM6 + PP3 + PP4
3	M	KMT2A	c.4219–2 A > T		exon 10 skipping	mis-splicing			*de novo*	hetero	novel	P	PVS1 + PM1 + PM2 + PM6 + PP3 + PP4
4	F	KMT2A	c.6217 A > T		p.Lys2073*	nonsense			NA	hetero	novel	P	PVS1 + PM1 + PM2 + PP3 + PP4
5	F	KMT2A				partial gene deletion (31 KB; exon 9–31)		grch37:11:118354408–118,385,822	NA	hetero	novel	p	PVS1 + PM1 + PM2 + PP3 + PP4
6	F	KMT2A	c.2881_2884del		p.Leu961*	nonsense			NA	hetero	novel	p	PVS1 + PM2 + PP3 + PP4
7	M	KMT2A	c.3619 C > T		p.Gln1207*	nonsense			NA	hetero	novel	P	PVS1 + PM2 + PP3 + PP4
8	F	KMT2A	c.2318dup		p.Ser774Valfs*12	frameshift			NA	hetero	previously reported	P	PVS1 + PM2 + PP3 + PP4
9	M	KMT2A	c.3497 A > G		p.Asp1166Gly	missense			*de novo*	hetero	novel	LP	PM1 + PM2 + PM5 + PM6 + PP3 + PP4
10	M	KMT2A	c.220del		p.Val74Phefs*76	frameshift			NA	hetero	novel	P	PVS1 + PM2 + PP3 + PP4

*NA* Not analyzed; *P* Pathogenic; *LP* Likely pathogenic

* Nucleotide numbering was based on the KMT2A cDNA sequence (NM_001197104.2) and genomic DNA sequence (grch37)

All except one (9/10) were novel, and no recurrent mutations were identified. Nine variants were classified as pathogenic, and the other one was likely pathogenic (LP) according to the ACMG classification. One missense variant, p.Asp1166Gly, could be classified as LP (PM1 + PM2 + PM5 + PM6 + PP3 + PP4) because the site at which this missense variant exists is the zinc finger containing the CXXC domain (PM1), which is thought to be essential for the KMT2A protein to bind to target genes [3]. A novel missense change at the amino acid residue, which was determined to be pathogenic, was previously observed (p.Asp1166Ala)(PM5) [14]. This variant was absent from the population databases (PM2) and was confirmed to be *de novo* via parental testing (PM6). Additionally, multiple in silico prediction tools supported the deleterious effects of variant (PP3), and the patient’s phenotype in its entirety is consistent with WSS (PP4). Sanger sequencing of the parents confirmed the *de novo* status in four patients.

## Discussion

Chromatinopathies are a group of genetic disorders characterized by mutations in the genes responsible for chromatin remodeling and transcriptional regulation. Among chromatinopathies, notable syndromes are Rubinstein-Taybi syndrome (RSTS), Kabuki syndrome (KS), and Cornelia de Lange syndrome (CdLS), which are respectively caused by mutations in *EP300* or *CREBBP* encoding histone acetyltransferases, *KMT2D* or *KDM6A* affecting histone methylation, and cohesion complex genes, which are crucial for transcriptional regulation [15–17]. WSS is also classified in this group of chromatinopathies, exhibiting overlapping phenotypes with RSTS, KS, and CdLS, including growth retardation, intellectual disability, and characteristic facial features [17–19]. It can be inferred that genetic abnormalities related to transcriptome disturbances may be associated with overlapping trait expression in these diseases. Although it is challenging to pinpoint a unique feature of WSS, hypertrichosis cubiti seems to be relatively rare in other chromatinopathies, as there are few reports of its occurrence in these conditions. However, it is observed in approximately 60% of patients [8], making it a rather distinctive phenotype for this condition.

The *KMT2A* gene is located on chromosome 11q23.3. Pathogenic variants of WSS can arise from any site along the entire gene without hot spots [20]. The types of reported pathogenic variants include missense, frameshift, nonsense, exonic deletion, and splice-site variants [1, 3]. Although not fully understood, some genotype-phenotype correlations have been suggested. According to Sheppard et al., participants with loss-of-function variants were more likely to have hypotonia, whereas those with non-loss-of-function variants were more prone to seizures [1]. Additionally, cases have been reported suggesting that missense mutations in the CXXC zinc finger domain may be associated with more profound neurodevelopmental outcomes [3, 10, 21].

It is difficult to target and test *KMT2A* in patients with WSS because it is not possible to select a representative and specific trait for this disease. Most patients are eventually diagnosed through extensive genetic testing such as CMA or ES. The patients included in this study were also diagnosed with WSS using ES (9/10) and CMA (1/10), and WSS was clinically suspected at the time of physical examination in patients diagnosed later. According to the literature, most subjects are diagnosed using ES, gene panels related to DD/ID, or genes related to CdLS or KS [1, 2, 22].

Among facial appearances, blepharoptosis was the most common characteristic in our patient cohort, accounting for four of 10 patients [1]. Though direct comparisons should be made with caution given the Limited sample size, this proportion was similar to that in the largest study of 104 patients, which was reported as 43%. In contrast, a 33 French cohort study reported ptosis in 16%, and another Chinese study, which compared phenotypes between the Chinese (14 patients) and French (33 patients) cohorts, demonstrated that the Chinese cohort had a higher frequency of ptosis than the French cohort [21]. Other common facial phenotypes suggested by relevant studies include a flat face, thick eyebrows, long eyelashes, wide nasal bridge, broad nasal tip, hypertelorism, and down-slanting palpebral fissures. Each of these features was also observed in our cohort; however, only a minority of patients had distinct and prominent findings, including thick or long eyebrows in two patients, flat nasal bridges in one, down-slanted palpebral fissures in one, and long eyelashes in one. Because we did not collect photographs of all patients, we could not recall facial appearances well or evaluate facial appearances in detail and had to rely on medical records.

Regarding growth profiles, nine of 10 patients had a history of failure to thrive, and seven of the patients showed short stature, which is consistent with previous reports. A history of feeding difficulties in infancy was recognized in four out of 10 patients; however, tube feeding was required in only one case. Reported rates of feeding difficulty in previous studies have ranged from 31 to 66% [1, 2, 21]. To date, there have been no large-scale studies analyzing final adult heights of patients with WSS, but in one case report, a female patient’s adult height at age 23 years was checked as 150.6 cm (4th percentile), and another female patient who was diagnosed with central precocious puberty reached a final adult height of 126.0 cm [9, 22]. Although none of the patients in our study had documented growth hormone deficiency, there are some reports that 50% of patients with WSS have been diagnosed with growth hormone deficiency [2, 11]. The exact and precise molecular mechanism by which short stature or growth hormone deficiency occurs has not been identified, but histone methylation may be linked to this phenotype, considering that growth hormone deficiency is often described in patients with Kabuki syndrome, another chromatinopathy.

Among those with documented data, the developmental or intellectual functioning of our patients belonged to ID class 3 (FSIQ 50–70). Very Limited data have been currently provided about the degree of ID in patients with WSS, but a previous study of 25 patients found that four (16%) patients had an FSIQ below 50, 10 (40%) patients had an FSIQ of 50–70, and 11 (44%) patients had an FSIQ above 70 [1]. Thus, the spectrum of the intellectual ability of the WSS is assumed to be relatively wide. This was also true in the present study, as the patients’ developmental achievements varied. One patient (C-01) still could not walk but only creep, even when she was almost 5 years old, while another patient (C-02) was able to conduct learning courses as her peers in a general class of an ordinary school with more assistance than her peers.

A notable point of our study regarding neurological characteristics was that microcephaly was observed in four patients. Microcephaly in patients with WSS was reported in several case reports, and different cohort studies came up with different values (33% in the French cohort and 50% in the Chinese cohort) [2, 7, 9, 21, 23]. The association between microcephaly and mental retardation has been widely recognized and studied. In one study, a statistically significant difference was reported between the normocephalic and microcephalic groups with respect to the presence or absence of normal intelligence or mental retardation [24]. An experimental report suggested that brain volume is related to fibroblast growth factor, which is essential for neurogenesis [25].

Abnormalities of the corpus callosum have been commonly observed on MRI in a number of reported cases [25–28]. This study identified corpus callosum abnormalities as the most prevalent neuroimaging finding, observed in two out of 8 patients. This observation is consistent with previous findings on WSS [1].

Another distinct medical issue in our patients was the presence of musculoskeletal problems, particularly finger or toe anomalies (6/10). The deformities included clinodactyly, brachydactyly, and syndactyly. Although the values varied between reports, ranging from 21 to 71%, finger/toe anomalies were not prominently described in the WSS because most of these were minor and were not clinically significant. The frequency of sacral dimples was consistent with that in the Literature, ranging from 25 to 50.5% [1]. A previous study reported that this could be a useful indicator for WSS diagnosis, especially in combination with ID [2]. Other orthopedic abnormalities included broad first digits, tapering fingers, long hallux, vertebral anomalies, and rib anomalies; however, these were not clinically noticeable in our cohort.

Hypertrichosis cubiti was once regarded as a pathognomonic sign of WSS, but in our cohort, one patient had hypertrichosis cubiti, which was Limited to the elbows, but in the other nine patients, hirsutism was observed in more than one body part other than the elbows. Therefore, it may be only a readily or commonly associated sign of WSS. In our cohort, all the patients had hypertrichosis. However it is difficult to say that all patients with WSS might show hypertrichosis, as the reported frequency of hypertrichosis was less than 70% in the cohort [1, 2].

Clinical signs of WSS vary; however, none of these symptoms or signs can directly indicate WSS, as they are not unique or specific to WSS. Therefore, it is important for clinicians to suspect WSS when a patient presents with a combination of these nonspecific abnormalities. This study had several limitations. First, it included a small number of patients, which may have limited the generalizability of the findings. Additionally, the retrospective design of the study may have inherent limitations such as potential bias and incomplete data. Furthermore, the lack of long-term follow-up data restricts our understanding of disease progression and outcomes in adulthood and later in life.

## Conclusion

This study identified postnatal growth retardation, ptosis, developmental delay/intellectual disability, microcephaly, digit anomalies, sacral dimples, and hypertrichosis as characteristic features of WSS in the largest Korean cohort to date. Our findings are consistent with those of previous studies on WSS. However, diagnosing WSS at first glance remains challenging because of the variable spectrum of developmental delays and the non-specific nature of many clinical features.

The diverse clinical presentations of WSS highlight the importance of increasing awareness among clinicians. Enhanced recognition will facilitate appropriate genetic testing for early diagnosis, enabling the proactive management and monitoring of potential complications. Additionally, it will support the provision of comprehensive genetic counseling to affected families.

## Data Availability

Data generated during the current study are available from the corresponding author, upon reasonable request. The datasets used for investigating protein domains of Histone-lysine N-methyltransferase 2 A are publicly available in the [UniProt] repository (https://www.uniprot.org/uniprotkb/Q03164/entry#family_and_domains).

## Abbreviations

ACMG: American College of Medical Genetics and Genomics
CdLS: Cornelia de Lange syndrome
CMA: Chromosomal microarray analysis
DD: Developmental delays
ES: Exome sequencing
FSIQ: Full-Scale Intelligence Quotient
ID: Intellectual disabilities
KS: Kabuki syndrome
LP: Likely pathogenic
MRI: Magnetic resonance imaging
NICU: Neonatal Intensive Care Unit
PDA: Patent ductus arteriosus
RSTS: Rubinstein-Taybi syndrome
WSS: Wiedemann-Steiner syndrome
WHO: World Health Organization
